# Influenza illness averted by influenza vaccination among school year children in Beijing, 2013‐2016

**DOI:** 10.1111/irv.12585

**Published:** 2018-07-01

**Authors:** Yi Zhang, Zhidong Cao, Valentina Costantino, David J. Muscatello, Abrar A. Chughtai, Peng Yang, Quanyi Wang, C. Raina MacIntyre

**Affiliations:** ^1^ Beijing Municipal Center for Disease Prevention and Control Institute of Infectious Diseases and Endemic Diseases Control Beijing China; ^2^ Beijing Research Center for Preventive Medicine Institute of Infectious Diseases and Endemic Diseases Control Beijing China; ^3^ School of Public Health and Community Medicine The University of New South Wales Sydney NSW Australia; ^4^ The State Key Laboratory of Management and Control for Complex Systems Institute of Automation Chinese Academy of Sciences Beijing China; ^5^ College of Public Service & Community Solutions and College of Health Solutions Arizona State University Tempe AZ USA

**Keywords:** influenza, models, students, vaccination

## Abstract

**Background:**

The benefit of school‐based influenza vaccination policy has not been fully addressed in Beijing.

**Objectives:**

To evaluate the benefit of school‐based influenza vaccination policy launched in Beijing.

**Methods:**

Using existing surveillance and immunization data, we developed a dynamic transmission model to assess the impact of influenza vaccination in school‐going children. The outcome was defined as the averted number of medically attended influenza illnesses and the prevented disease fraction to all children aged 5‐14 years for the 2013/14, 2014/15, and 2015/16 seasons.

**Results:**

We estimated that during the three consecutive influenza seasons, the averted number of medically attended influenza illnesses among children aged 5‐14 years was around 104 000 (95% CI: 101 000‐106 000), 23 000 (95% CI: 22 000‐23 000), and 21 000 (95% CI: 21 000‐22 000), respectively. Corresponding prevented fractions to all children aged 5‐14 years were 76.3%, 38.5%, and 43.9%.

**Conclusions:**

In Beijing, school‐based vaccinations reduced a substantial number of medically attended influenza illnesses despite seasonal variation in the prevented fraction. This is strong supportive evidence for the continuation of school‐based vaccination programs to reduce the influenza burden in this age group.

## 1 INTRODUCTION

Influenza causes substantial morbidity and mortality all over the world. The World Health Organization (WHO) recommended that populations at higher risk for complications and hospitalizations should be vaccinated.^1^ Healthy school‐going children, although excluded from the defined vulnerable populations, are at a higher risk of contracting the influenza virus because of the increased probability of transmission due to more frequent close contact within this age group.^2^ School‐based influenza vaccination programs have therefore been recommended in some settings as an extension of existing influenza vaccination programs but remain rare globally.^3^, ^4^ In Beijing, in 2009, a free influenza vaccination policy was launched in primary, secondary, and high schoolchildren who are 6‐18 years old.^5^ Temporary points of vaccination (POVs) are established at schools, and students with their parent's written consent are organized to receive the vaccination there. Moreover, students are able to receive the free vaccination at community POVs located if the temporary school POVs are closed. Despite 8 years of a funded school‐based influenza vaccine policy, there has not been any research evaluating the impact of this vaccination policy.

In recent years, several studies assessed vaccine effectiveness (VE) in Beijing residents to demonstrate the benefit of influenza vaccination. Moreover, to understand the public health benefit of school‐based vaccination policy, a long‐term ecological overview study based on school outbreak data was conducted. The findings indicated that the reduction in school outbreaks by vaccination was significantly dependent on vaccination coverage (VC) and VE.^6^ However, the finding was not quantitative. Of the existing data on VE and the reduction in outbreaks, the impact of the school‐based vaccination policy has not yet been fully evaluated. New approaches to quantifying cases and medical encounters averted by vaccination are needed to demonstrate the impact of school‐based vaccination policy.

In this study, using a transmission dynamic model and judging by the change of transmission intensities and transmission patterns with vs without vaccination, we estimated the averted number of medically attended (including outpatient and emergency attended) influenza illnesses and prevented fractions in schoolchildren from the 2013‐2016 seasons. These data can directly show the gains of the vaccination policy to stakeholders and inform future policymaking.

## 2 METHODS

### 2.1 Methods overview

In this study, the target outcome is the reduced number of influenza‐associated medically attended illnesses among schoolchildren aged 5‐14 years. The target age group was 6‐18 years; however, we were only able to obtain data on population size and medical attendances in the age group of 5‐14 years. The outcome was estimated in several steps. First, we estimated the number of influenza infections in Beijing among children aged 5‐14 years using surveillance data. Then, the number of influenza infections was used in a susceptible‐exposed‐infectious‐removed (SEIR) model to simulate transmission dynamic processes accounting for VC and VE. During this process, a series of parameters of the SEIR model were estimated. Next, the parameters were again used in the SEIR model to simulate the number of influenza infections without vaccination. The difference between the estimated numbers of influenza infections with vs without vaccination equals the influenza infections averted by vaccination. Lastly, the averted number of influenza‐associated medically attended illnesses was calculated.

### 2.2 Data sources

#### 2.2.1 Number of influenza infections

To determine the number of influenza illnesses averted, we needed an estimate of the total number of influenza infections in the Beijing population in children aged 5‐14 years in each season. To do this, we progressively scaled up the number of infections detected by influenza‐like‐illness (ILI) surveillance, accounting for underreporting in ILI surveillance, as shown in the published study.^7^

The number of infections detected by ILI surveillance was calculated using the number of ILI cases reported by hospitals, multiplied by the proportion of ILI cases that were positive for influenza in each week. ILI surveillance is conducted in outpatient and emergency departments in all 421 general hospitals in Beijing, reporting for weekly number of ILI cases by age groups. ILI is defined as a person presenting with fever (temperature >38°C) and cough or sore throat in the absence of other diagnosis. The ILI surveillance is enhanced through the collection of virological data from 24 sentinel hospitals, where the age group specific weekly rates of influenza among ILI cases were acquired. In this study, influenza‐associated medically attended illness is defined as the number of infections detected by ILI surveillance.

We scaled up the number of influenza‐associated medically attended illnesses among children aged 5‐14 years to estimate the number of influenza infections, in reference to the published methods and parameters.^7^ It is estimated that every influenza‐associated medically attended illness represented 9.08 (95% CI: 7.21‐12.21) influenza infections in the Beijing population aged 5‐14 years old.

#### 2.2.2 Influenza vaccine coverage

We defined the cumulative VC for influenza as the proportion of population vaccinated. In Beijing, only trivalent inactivated vaccine (TIV) is administered. Seasonal influenza vaccines are offered from the date of 15 October each year. The influenza year was defined as from 15 October to 14 October of the following year. The weekly number of influenza vaccinations among children aged 5‐14 years was obtained from an online registration system called Beijing Immunization Management Information System. The seasonal cumulative VC was 49%, 45%, and 43% in children aged 5‐14 years in the 2013/14, 2014/15, and 2015/16 seasons, respectively.

#### 2.2.3 Influenza VE

We reviewed observational studies with VEs in the previous three flu seasons by Beijing CDC^8^, ^9^, ^10^, ^11^ and found that the VE against medically attended illness for children aged 5‐14 years was not always reported. Instead, we used the VE estimates that are either from the outpatients or subjects closest in age. Therefore, the VE values used in the model were 59.5% for influenza A (H1N1) in the 2009 season, 59.5% for influenza A (H3N2), 42.4% for influenza B in the 2013‐14 season, and 27.9% for influenza A (H3N2) in the 2014‐15 season.^8^ The published data showed vaccine presented no effectiveness against influenza B in the 2014‐15 season and influenza A and B in the 2015‐16 season;^8^, ^11^ therefore, we used a low effectiveness of 20% instead. As estimates of VE varied across studies for the same season, sensitivity analyses were performed to reflect the uncertainty of the assumptions, where the VE was assumed to be 10% lower and 10% greater, respectively, on the base of original ones in all seasons.

### 2.3 Calculation of outcome

#### 2.3.1 Structures of SEIR model

The SEIR model can provide a basic description of the transmission dynamics of influenza using a simple parameterized set of ordinary differential equations. The total number of individuals in the population at time *t* is N = S(*t*) + E(*t*) + I(*t*) + R(*t*), where S(*t*) represents the susceptible individuals without prior immunity at time *t* = 1, E(*t*) are the infected in latent state, before onset illness (2), I(*t*) are the symptomatic infected (3), and R(*t*) is made up of individuals that have either recovered from the disease or gained immunity from vaccination (4). Equations (1), (2), (3), (4) of the SEIR model are given as

(1) $$\frac{dS(t)}{\mathrm{dt}}=-\beta \left(t\right)\frac{S(t)\cdot I(t)}{N}$$

(2) $$\frac{dE(t)}{\mathrm{dt}}=\beta \left(t\right)\frac{S(t)\cdot I(t)}{N}-\sigma \cdot E\left(t\right)$$

(3) $$\frac{dI(t)}{\mathrm{dt}}=\sigma \cdot E\left(t\right)-\gamma \cdot I\left(t\right)$$

(4) $$\frac{dR(t)}{\mathrm{dt}}=\gamma \cdot I\left(t\right)$$

(5) $$\beta \left(t\right)=\left\{\begin{array}{cc} \beta_{1}, & t\leq T_{1}\\ \beta_{2}, & T_{1}<t\leq T_{2}\\ \beta_{3}, & t>T_{2} \end{array}\right.$$

β(*t*) is the transmission coefficient, representing the probability of transmission per contact between an infected and a susceptible individual. As influenza transmission intensity changed during an epidemic season and the duration of each intensity varied,^12^ we assumed different values of β corresponding to three different infectious times in this study. σ is the rate at which an exposed individual becomes infectious per unit of time. γ stands for the rate at which an infectious individual recover per unit of time. σ and γ are the inverses of the latent and infectious periods, respectively.

Vaccination was given to target populations; therefore, the component of N_vac_ was introduced into the SEIR model, where N_vac_ (*t*) stands for the number of people vaccinated at time *t*. A successfully vaccinated person is considered immunized after 2 weeks. Vaccination reduces the number of susceptible individuals, through acquired immunization, as well as the number of infectious contacts, through herd immunity. The impact of vaccination depends on VC and VE (*k*). The model was built by MATLAB (trial version).

(6) $$\frac{dS(t)}{\mathrm{dt}}=-\beta \left(t\right)\frac{\left(S\left(t\right)-N_{\mathrm{vac}}\left(t-2\right)k\right)\cdot I\left(t\right)}{N}-N_{\mathrm{vac}}\left(t-2\right)k$$

(7) $$\frac{dE(t)}{\mathrm{dt}}=\beta \left(t\right)\frac{\left(S\left(t\right)-N_{\mathrm{vac}}\left(t-2\right)k\right)\cdot I\left(t\right)}{N}-\sigma \cdot E\left(t\right)$$

(8) $$\frac{dI(t)}{\mathrm{dt}}=\sigma \cdot E\left(t\right)-\gamma \cdot I\left(t\right)$$

(9) $$\frac{dR(t)}{\mathrm{dt}}=\gamma \cdot I\left(t\right)+N_{\mathrm{vac}}\left(t-2\right)k$$

$$\beta \left(t\right)=\left\{\begin{array}{cc} \beta_{1}, & t\leq T_{1}\\ \beta_{2}, & T_{1}<t\leq T_{2}\\ \beta_{3}, & t>T_{2} \end{array}\right.$$

### 2.4 Averted number of influenza infections and averted number of influenza‐associated medically attended illness

We first simulated the curve of the number of influenza infections with vaccination by fitting the model (formula (6), (7), (8), (9)) to initial data, and parameters including σ, γ, β_1_, β_2_, β_3_, *T*_1_, and *T*_2_ were solved using Runge‐Kutta methods during this process. We ran the fitting program for 100 times, and 95% confidence interval of each parameter was obtained. The estimated number of influenza infections with vaccination and its 95% CI was then obtained by adopting the parameters into the SEIR model. Afterward, we estimated the number of influenza infections for a scenario without vaccination by the SEIR model (formula (6), (7), (8), (9), VE = 0). Averted number of influenza‐associated medically attended illnesses was defined as the averted number of influenza infections by vaccination divided by 9.08 (see 2.2.1). The prevented fraction was defined as the number of averted influenza infections divided by the total influenza infections that would have been expected in an unvaccinated population.

## 3 RESULTS

### 3.1 Estimated transmission dynamics

From the 2013/14 to the 2015/16 season, σ was estimated at 1.6 on average (range: 0.7‐3.2), where the latent period is around 4.4 days on average (range: 2.2‐9.7); γ was estimated at 0.8 on average (range: 0.6‐1.2), where the infectious period is 8.6 days (range 6.1‐12.5) correspondingly. The mean estimated *R*_0_ was 1.3 (range: 0.7‐1.8) over the three seasons (Table 1). The estimated model parameters from epidemiological data by subtype, season, and epidemic period are shown in Table S1.

**Table 1:** Estimations of latent period, infectious period, and average basic reproduction numbers from epidemiological data by subtypes and by seasons

Parameters	Interpretation	2013/14 season			2014/15 season		2015/16 season			
		H1	H3	B/Yamagata	H3	BY	H1	H3	B/Victoria	B/Yamagata
1/σ	Latent period	4.0 (3.8, 4.2)	5.1 (4.8, 5.5)	9.7 (9.0, 10.6)	4.0 (3.8, 4.2)	5.0 (4.6, 5.4)	3.1 (3.0, 3.3)	5.7 (5.4, 6.1)	6.1 (5.8, 6.5)	2.2 (2.1, 2.3)
1/γ	Infectious period	12.5 (11.7, 13.5)	9.7 (9.2, 10.1)	8.5 (8.3, 8.9)	9.9 (9.2, 10.6)	8.8 (8.4, 9.1)	8.3 (8.0, 8.8)	7.8 (7.5, 8.0)	8.2 (8.0, 8.5)	6.1 (6.0, 6.4)
*R*_0_ a	Basic reproduction number	1.8 (1.7, 1.9)	1.5 (1.5, 1.6)	1.7 (1.6, 1.7)	1.5 (1.4, 1.5)	1.5 (1.4, 1.5)	0.8 (0.8, 0.9)	0.9 (0.9, 0.9)	1.2 (1.1, 1.2)	0.7 (0.6, 0.7)

a *R*_0_ is the average *R*_0_ estimations across the three phases of an epidemic season.

### 3.2 Averted number of influenza‐associated medically attended illnesses and prevented fraction

**Figure 1:**
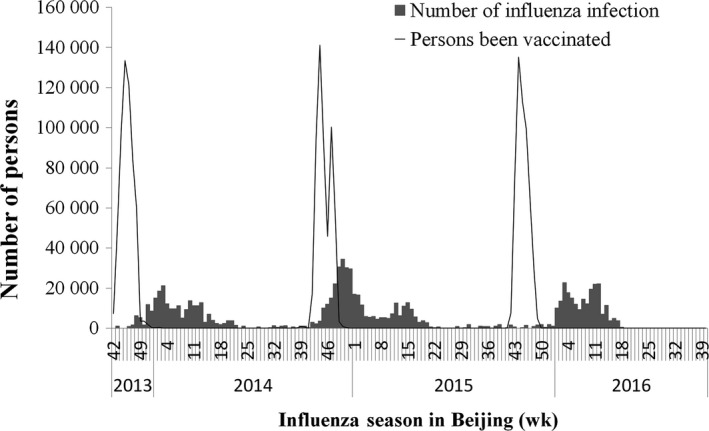
Estimated Number of Influenza Infections and Registered Number of Persons Been Vaccinated in 2013/14, 2014/15, and 2015/16 Seasons in Beijing

**Figure 2:**
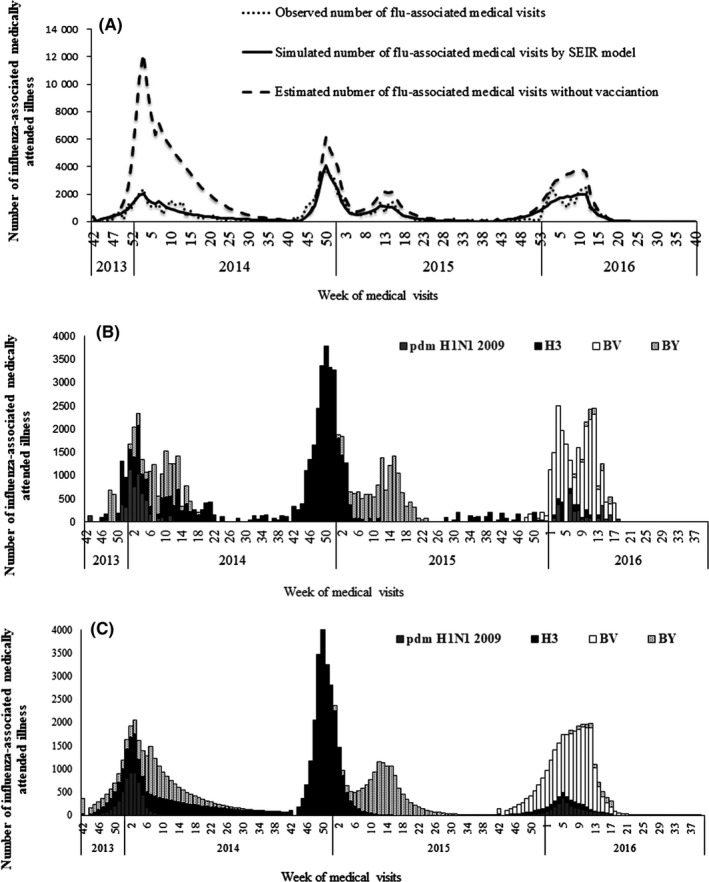
A, Number of Influenza‐Associated Medically Attended Illness With and Without Vaccination Estimated by SEIR Model in 2013/14, 2014/15, and 2015/16 Seasons; B, Observed Number of Influenza‐Associated Medically Attended Illness; C, Simulated Number of Influenza‐Associated Medically Attended Illness

The weekly number of persons vaccinated and the number of estimated influenza infections among children aged 5‐14 years from 2013 to 2016 are shown in Figure 1. Influenza vaccination averted approximately 104 000 (CI: 101 000‐106 000), 23 000 (CI: 22 000‐23 000), and 21 000 (CI: 21 000‐22 000) outpatient and emergency visits among children aged 5‐14 years in Beijing, in the 2013/14, 2014/15, and the 2015/16 seasons, respectively. The simulated number of attended influenza illnesses with and without vaccination is shown in Table 2 and Figure 2. The prevented fraction to all children aged 5‐14 years was 76.3%, 38.5%, and 43.9% in the 2013/14, 2014/15, and 2015/16 seasons, respectively (Table 2).

**Table 2:** Predicted impact of vaccinations among children 5‐14 y, in 2013/14, 2014/15, 2015/16 season

	2013/14	2014/15	2015/16
Number of vaccinations	565 400	550 300	528 179
Vaccination coverage rate	49%	45%	43%
Simulated number of influenza infections	259 671 (258 570, 260 771)	3 328 311 (325 949, 330 674)	246 808 (245 851, 247 765)
Estimated number of influenza infections without vaccine	1 232 172 (1 205 082, 1 259 261)	534 306 (527 478, 541 134)	440 574 (433 685, 447 465)
Number of influenza infection averted	939 897 (913 688, 966 106)	205 758 (199 421, 212 095)	193 582 (186 914, 200 249)
Averted number of influenza‐associated medically attended illness	103 513 (100 626, 106 399)	22 660 (21 963, 23 358)	21 320 (20 585, 22 054)
Prevented fraction	76.3% (75.8%, 76.7%)	38.5% (37.8%, 39.2%)	43.9% (43.1%, 44.8%)

We estimated the number of infections averted by direct and indirect vaccine effects (Table S2). The results showed that 3.7%, 13.8%, and 4.8% of infections were averted by indirect vaccine effects in 2013/14, 2014/15, and 2015/16 season, respectively.

### 3.3 Sensitivity analysis

To reflect the uncertainty of this result, we performed a sensitivity analysis where VE was assumed to be 10% lower and 10% greater in all seasons, while VC was unchanged. Results of this sensitivity analysis are shown in Table 3. It is shown that the benefit of vaccination increased with improving VE. The overall averted number of influenza‐associated medical visits were around 81 000, 12 000, and 9000 in three seasons, respectively, when VE decreased by 10%; and 137 000, 38 000, and 35 000 in three seasons, respectively, when VE increased by 10%.

**Table 3:** Sensitivity analysis: averted number of influenza‐associated medical visits and prevented fractions under different level of VE hypothesis and different population size hypothesis

	Averted number of influenza‐associated medical visits	Prevented fraction
VE decreased by 10%^a^		
2013/14 season	80 879 (77 056, 84 702)	74.0% (73.1%, 75.0%)
2014/15 season	12 361 (11 694, 13 028)	25.5% (24.6%, 26.3%)
2015/16 season	8967 (8509, 9425)	25.1% (24.2%, 25.9%)
VE increased by 10%^b^		
2013/14 season	137 017 (130 783, 143 252)	82.6% (52.1%, 83.1%)
2014/15 season	38 144 (36 596, 39 693)	51.2% (50.3%, 52.1%)
2015/16 season	35 154 (33 841, 364 68)	56.5% (55.7%, 57.4%)
Larger population size^c^		
2013/14 season	125 660 (121 063, 130 257)	76.8% (76.2%, 77.4%)
2014/15 season	27 978 (26 794, 29 163)	36.9% (36.0%, 37.8%)
2015/16 season	25 547 (24 414, 26 680)	41.6% (40.6%, 42.7%)
Smaller population size^d^		
2013/14 season	96 203 (91 671, 100 736)	80.7% (80.1%, 80.2%)
2014/15 season	18 102 (17 155, 19 050)	38.6% (37.4%, 39.8%)
2015/16 season	17 369 (16 327, 18 412)	44.5% (43.2%, 45.9%)

a VE was 49.5% for H1, 49.5% for H3, 32.4% for BY in 2013/14 season; VE was 17.9% for H3, 10% for BY in 2014/15 season; VE was 10% for H1, H3, BY, BV in 2015/16 season.

b VE was 69.5% for H1, 69.5% for H3, 52.4% for BY in 2013/14 season; VE was 37.9% for H3, 30% for BY in 2014/15 season; VE was 30% for H1, H3, BY, BV in 2015/16 season.

c Larger population size: every influenza‐associated medically attended illness represented 12.21 influenza infections in population aged 5‐14 y. The number of infections was 322 969, 488 391, and 314 665 in the 2013/14, 2014/15, and 2015/16 seasons, respectively.

d Smaller population size: every influenza‐associated medically attended illness represented 7.21 influenza infections in population aged 5‐14 y. The number of infections was 190 713, 288 395, and 187 216 in the 2013/14, 2014/15, 2015/16, seasons, respectively.

We performed a sensitivity analysis using different influenza infection estimations to evaluate the impact of the number of influenza infections on the estimation of the prevented fraction among all children aged 5‐14 years. The results showed that the higher estimation of influenza infections resulted in a lower prevented fraction, and vice versa, but the impact was small (Table 3).

## 4 DISCUSSION

We used a dynamic transmission model to estimate the benefit of influenza vaccination in children aged 5‐14 years for three consecutive seasons, by estimating the averted number of influenza illnesses and related medical visits. The findings showed that influenza vaccination could prevent a substantial number of medical visits in this age group in Beijing. The results support the current policy of funded vaccination for schoolchildren and emphasize the importance of improving vaccine coverage to reduce the influenza burden.

Schoolchildren are one of only two groups of the population in Beijing eligible for free influenza vaccination, due to the increased potential for infection transmission among this group. They are, however, not the top prioritized population for vaccination recommended by either the WHO or China Center for Diseases Prevention and Control (China CDC), as school‐going children are not at higher risk of severe outcome of influenza infection than others (the elderly, individuals with medical condition, pregnant women). Our study showed that vaccination could avert a substantial number of medical visits, especially in years with good VE. However, in years with suboptimal VE, for example, a 10% VE in the 2015/16 season as shown in the sensitivity analysis, a sizable number of medically attended influenza illnesses could still be prevented.^13^ Our results suggested that vaccination can effectively prevent influenza illness among schoolchildren, even with mismatched vaccine.^10^ Schoolchildren is a group that is easy to reach, and feasible to vaccinate, when compared with other vulnerable populations. Given these features, the current policy that encourages influenza vaccine uptake among schoolchildren by providing free vaccination should be supported.

Our study suggested that VE has a strong impact on vaccination outcome, and a small improvement of VE will result in a significant reduction in cases. The timing of vaccination relative to disease occurrence also affects the benefit of vaccination.^14^ Comparing the 2014/15 season with the 2015/16 season, the fraction number was significantly lower in 2014/15, while the coverage rate and VE were higher in 2014/15. On the one hand, this is because the influenza activity was higher in the 2014/15 than the 2015/16 season, as shown in Table 1. On the other hand, we noticed that in the 2014/15 season the influenza epidemic occurred earlier, meaning that vaccination occurred later relative to the onset of the season (Figure 2). Both our finding and a previous study^14^ suggested that an early schedule for vaccination of schoolchildren might yield a greater fraction. Based on these evidences, we moved up the start‐up vaccination date to September 20 since 2017, that is, 25 days ahead of the previous vaccination dates from 2009 to 2016.

In our study, the VC was similar over years, so the impact of VC was not evaluated. In the United States, CDC estimated the averted number of influenza‐associated outcomes by vaccination in consecutive seasons by age group, and the results suggested that a greater fraction of disease was prevented as greater fractions of the population were vaccinated.^14^ It is estimated that a 20% increase in VC among schoolchildren corresponds to an 8% decrease in emergency department visits.^15^ More effort is therefore needed to improve coverage among schoolchildren in the future.

Our study has some limitations. Firstly, we only assess the impact of influenza vaccination among children aged 5‐14 years, while schoolchildren are generally 6‐18 years of age. Thus, the number of influenza‐associated medically attended illnesses averted by vaccinating all schoolchildren is underestimated in this study. Secondly, we assumed an enclosed population that only included children aged 5‐14 years old. However, the school‐based vaccination policy not only aims at reducing morbidity in this age group, it is hoped that it would result in reduced transmission in other age groups, as schoolchildren may play a large role in the spread of influenza in the household and community.^16^ Therefore, the benefit of the policy may have been underestimated in our study, and an age‐structured model may better quantify the overall effect in the future. However, as mathematical modeling has never before been used to assess the benefit of the vaccination policy in Beijing, our study makes an important step forward in closing the knowledge gaps. Thirdly, as the epidemic data fluctuated, the fitness between model and data was suboptimal and the confidential intervals of estimated parameters were relatively large. But generally, the parameter estimations are in accordance with their corresponded epidemiological meanings. Fourth, the number of influenza infections was estimated based on ILI surveillance and virological surveillance. The influenza positive rate among ILI cases may have been overestimated if physicians unconsciously swabbed cases they clinically felt were more likely to have influenza after applying the criteria for ILI. Although of this possibility, the sensitivity analysis showed that the overestimation of the number of influenza infections has a very small impact to the prevented fraction estimation. Five, there is a possible that persons who are more likely to get influenza are also more likely to get vaccinated, and therefore leading to a decreased VE estimation. If so, we may underestimate the impact of vaccination. However, this is less likely in 5‐ to 14‐year‐old children than in adults with chronic diseases. Moreover, in this study, we assumed that vaccinated and unvaccinated individuals have the same chance of exposure to infection. However, a school‐based study in the United States indicated that unvaccinated individuals may be in contact more often with other unvaccinated individuals than with vaccinated individuals, and this may counteract herd immunity to some extent.^17^ If the contact patterns in China are similar to those in the United States and this effect is present, our results may somewhat overestimate the impact of vaccination. Lastly, preexisting immunity in school‐age children was not considered in this study due to a lack of reference data.

We used a dynamic transmission model to estimate the gains of the vaccination program, considering the influenza attack rate over times and the herd immunity effect. Furthermore, the immunization rates were obtained from the registration system rather than a sampled survey. The influenza surveillance data covered all general hospitals in Beijing, improving the representativeness of the database. Moreover, we estimated the season‐, subtype‐ and stage‐specific transmission parameters, considering different transmission periods, allowing a more complete estimate of the value of influenza vaccination in Beijing. We used prevented fraction to show the impact of vaccination policy. The index has been used by researches from CDC,^18^ and in a similar modeling study.^14^ Unlike absolute numbers of averted outcomes, which are associated with the influenza attack rate, the prevented fraction is mainly affected by VE and VC, making it a good method for evaluating impact of vaccination policy.

## 5 CONCLUSIONS

Vaccination against influenza among schoolchildren has a substantial impact on reductions in medical encounters for influenza in Beijing. The study indicates that improvement in VE and coverage will lead to greater protection. This approach could be used to estimate the averted number of influenza‐associated hospitalizations and death, while our estimation could be used to analyze the cost‐effectiveness of the free‐of‐charge policy.
