# Supplementary material for: Influenza illness averted by influenza vaccination among school year children in Beijing, 2013‐2016
Source: Influenza Other Respir Viruses. 2018 Jul 1;12(6):687–94. doi: 10.1111/irv.12585 (PMC6185895; doi:10.1111/irv.12585)
Supplement: Supplementary file 1 [file IRV-12-687-s001.docx]

Supplementary table1 Model parameters estimated from epidemiological data

| Parameters | Interpretation | Values | | | | | | | |  |
| --- | --- | --- | --- | --- | --- | --- | --- | --- | --- | --- |
|  |  | 2013/14 | | | 2014/15 | | 2015/16 | | | |
|  |  | H1 | H3 | BY | H3 | BY | H1 | H3 | BV | BY |
| σ | The rate at which an exposed individual becomes infected per unit of time | 1.76  (1.68,1.84) | 1.36  (1.27,1.45) | 0.72  (0.66,0.78) | 1.75 (1.65,1.86) | 1.40  (1.30,1.51) | 2.23  (2.09, 2.37) | 1.22  (1.14,1.30) | 1.15 (1.08,1.21) | 3.16 (3.01,3.30) |
| γ | The rate at which an infected individual recover per unit of time | 0.56 (0.52,0.60) | 0.72 (0.69,0.76) | 0.82 (0.79,0.84) | 0.71  (0.66,0.76) | 0.80  (0.77,0.83) | 0.84  (0.80,0.88) | 0.90  (0.87,0.93) | 0.85 (0.82,0.88) | 1.14 (1.10,1.17) |
| β_1*_ | Transmission coefficient | 1.87 (1.82,1.92) | 1.67 (1.61,1.73) | 1.05 (1.02,1.09) | 1.94  (1.88,2.00) | 1.57  (1.50,1.63) | 1.56  (1.51,1.61) | 1.28  (1.25,1.31) | 1.41 (1.36,1.45) | 1.38 (1.34,1.42) |
| β_2*_ | Transmission coefficient | 0.75 (0.69,0.81) | 0.59 (0.51,0.66) | 2.34 (2.20,2.47) | 0.81  (0.76,0.86) | 1.36  (1.30,1.42) | 0.70  (0.66,0.75) | 0.94  (0.89,1.00) | 1.16  (1.19,1.23) | 0.21 (0.18,0.25) |
| β_3*_ | Transmission coefficient | 0.17 (0.15,0.19) | 1.01  (0.95,1.07) | 0.72 (0.69,0.76) | 0.31  (0.27,0.35) | 0.54  (0.50,0.58) | 0.15  (0.12, 0.18) | 0.27  (0.24,0.30) | 0.34  (0.31,0.36) | 1.07  (0.86,1.28) |
| R0_1*_ | Basic Reproduction Number | 3.71 (3.49,3.93) | 2.33  (2.27,2.40) | 1.29 (1.26,1.33) | 2.84  (2.70,2.99) | 1.99  (1.91,2.07) | 1.89  (1.84, 1.95) | 1.43  (1.41,1.45) | 1.66  (1.63,1.69) | 1.21  (1.20,1.22) |
| R0_2*_ | Basic Reproduction Number | 1.40  (1.30,1.50) | 0.80  (0.70,0.90) | 2.87  (2.71,3.03) | 1.15  (1.11,1.19) | 1.73  (1.65,1.81) | 0.83  (0.82, 0.84) | 1.04 (1.02,1.07) | 1.40  (1.39,1.41) | 0.19  (0.16,0.21) |
| R0_3*_ | Basic Reproduction Number | 0.28 (0.26,0.30) | 1.40 (1.39,1.41) | 0.88  (0.85,0.91) | 0.42  (0.38,0.46) | 0.67  (0.63,0.72) | 0.18  (0.15,0.21) | 0.30 (0.27,0.33) | 0.39  (0.36,0.42) | 0.93  (0.76,1.11) |
| T1 | The week of β change | 12 | 13 | 12 | 8 | 18 | 16 | 14 | 14 | 10 |
| T2 | The week of β change | 15 | 16 | 17 | 12 | 24 | 28 | 21 | 23 | 23 |

* We assumed three different infectious times in each of the epidemic season. β1,β2, and β3 were the corresponding transmission coefficient, while R0_1_, R0_2_, and R0_3_ were the corresponding basic reproduction number of the three phrases.
