# Supplementary material for: Influenza illness averted by influenza vaccination among school year children in Beijing, 2013‐2016
Source: Influenza Other Respir Viruses. 2018 Jul 1;12(6):687–94. doi: 10.1111/irv.12585 (PMC6185895; doi:10.1111/irv.12585)
Supplement: Supplementary file 2 [file IRV-12-687-s002.docx]

Supplementary table2 : Number of infections averted by direct and indirect vaccine effect

| Season | Sub-types | Number of infections : without vaccine | Number of infections : with direct vaccine effect  ^*^ | Number of infections : with direct and indirect vaccine effect^$^ | Number of infections averted by direct effect | Number of infections averted by indirect effect |
| --- | --- | --- | --- | --- | --- | --- |
|  |  | (a) | (b) | (c) | (d=a-b) | (e=b-c) |
| 2013/14 | H1 | 302032 | 65021 | 50248 | 237011 | 14773 |
|  | H3 | 600206 | 124933 | 108520 | 475273 | 16413 |
|  | BY | 329934 | 105877 | 100904 | 224057 | 4973 |
|  | Total | 1232172 | 295831 | 259671 | 936341 | 36160 |
| 2014/15 | H3 | 333212 | 240125 | 219817 | 93087 | 20308 |
|  | BY | 201094 | 116594 | 108495 | 84500 | 8099 |
|  | Total | 534306 | 356719 | 328311 | 177587 | 28408 |
| 2016/17 | H1 | 47339 | 24848 | 22806 | 22491 | 2042 |
|  | H3 | 32909 | 18940 | 18063 | 13969 | 877 |
|  | BV | 345284 | 203624 | 197964 | 141660 | 5660 |
|  | BY | 13370 | 8706 | 8031 | 4664 | 675 |
|  | Total | 438902 | 256118 | 246864 | 182784 | 9254 |

* This is estimated using the following formulas:

$$\frac{\text{dS}\left( \text{t} \right)}{\text{dt}}\text{=-β}\left( \text{t} \right)\text{.}\frac{S(t)\cdot I\left( t \right)}{\text{N}}\text{-}\text{N}_{\text{vac}}\left( \text{t-2} \right)\text{k }$$

$$\frac{dE(t)}{dt}=\beta\left( t \right)\cdot\frac{S(t)\cdot I\left( t \right)}{N}-\sigma\cdot E(t)$$

$$\frac{dI(t)}{dt}=\sigma\cdot E\left( t \right)-\gamma\cdot I(t)$$

$$\frac{dR(t)}{dt}=\gamma\cdot I\left( t \right)+N_{vac}\left( t-2 \right)k$$

^$^ This is estimated using formula 6-10 in the main text
